# Highly Efficient Electrochemical Hydrogen Evolution Reaction at Insulating Boron Nitride Nanosheet on Inert Gold Substrate

**DOI:** 10.1038/srep32217

**Published:** 2016-08-25

**Authors:** Kohei Uosaki, Ganesan Elumalai, Hung Cuong Dinh, Andrey Lyalin, Tetsuya Taketsugu, Hidenori Noguchi

**Affiliations:** 1 Center for Green Research on Energy and Environmental Materials and Global Research Center for Environment and Energy based on Nanomaterials Science (GREEN), National Institute for Materials Science (NIMS), Tsukuba 305-0044, Japan; 2 International Center for Materials Nanoarchitectonics (WPI-MANA), National Institute for Materials Science (NIMS), Tsukuba 305-0044, Japan; 3 Graduate School of Chemical Sciences and Engineering, Hokkaido University, Sapporo 060-0810, Japan; 4 Department of Chemistry, Faculty of Science, Hokkaido University, Sapporo 060-0810, Japan

## Abstract

It is demonstrated that electrochemical hydrogen evolution reaction (HER) proceeds very efficiently at Au electrode, an inert substrate for HER, modified with BNNS, an insulator. This combination has been reported to be an efficient electrocatalyst for oxygen reduction reaction. Higher efficiency is achieved by using the size controlled BNNS (<1 μm) for the modification and the highest efficiency is achieved at Au electrode modified with the smallest BNNS (0.1–0.22 μm) used in this study where overpotentials are only 30 mV and 40 mV larger than those at Pt electrode, which is known to be the best electrode for HER, at 5 mAcm^−2^ and at 15 mAcm^−2^, respectively. Theoretical evaluation suggests that some of edge atoms provide energetically favored sites for adsorbed hydrogen, i.e., the intermediate state of HER. This study opens a new route to develop HER electrocatalysts.

Hydrogen is considered to be the cleanest fuel and a recent announcement of a commercial fuel cell car opened a new era of hydrogen-based economy. One must, however, realize that although CO_2_ is not emitted when hydrogen is used as a fuel, it is emitted during the hydrogen production since current hydrogen source is fossil fuels such as natural gas. Therefore, for hydrogen to be truly a clean fuel, it must be generated from water using renewable energy. Water electrolysis combined with solar- or wind-generated electricity is one of the most promising processes to realize sustainable energy system^1^. Electrochemical hydrogen evolution reaction (HER) has been studied for long time both theoretically and experimentally^2^,^3^,^4^,^5^,^6^,^7^,^8^,^9^. Although platinum group metals and their alloys are known to be the most efficient electrocatalysts for HER, for water electrolysis to become a practical hydrogen production process, cost reduction is essential and the use of expensive and less abundant Pt must be avoided. Recently inorganic catalysts such as nano-sized metal dichalcogenides such as MoS_2_ and WS_2_, which are semiconductors with layered structure, have been found to have high activity for HER and have drawn great attention due to their low cost and high chemical stability^10^,^11^,^12^,^13^,^14^.

We have recently reported that insulating hexagonal boron nitride (h-BN) nanosheet on inert gold substrate (BNNS/Au) acts as an electrocatalyst for oxygen reduction reaction (ORR)^15^,^16^,^17^. h-BN monolayer has similar geometric structure to that of graphene but it is an insulator with a wide band gap (˜5.8 eV)^18^. It is, however, theoretically demonstrated the band gap of h-BN monolayer can be considerably reduced if some defects are introduced and/or it is placed on metal substrates^19^,^20^ and it is experimentally shown that the atomically thin h-BN nanoribbons become semiconducting^21^ similar to metal dichalcogenides. Furthermore, hydrogen adsorption/storage ability of BN has been experimentally demonstrated^22^,^23^. Thus, considering the reasonably high activity for ORR, hydrogen adsorption activity, and similarity in structure with HER active metal dichalcogenides, one may expect BNNS to be an effective electrocatalyst for HER.

## Results and Discussion

**Figure 1:**
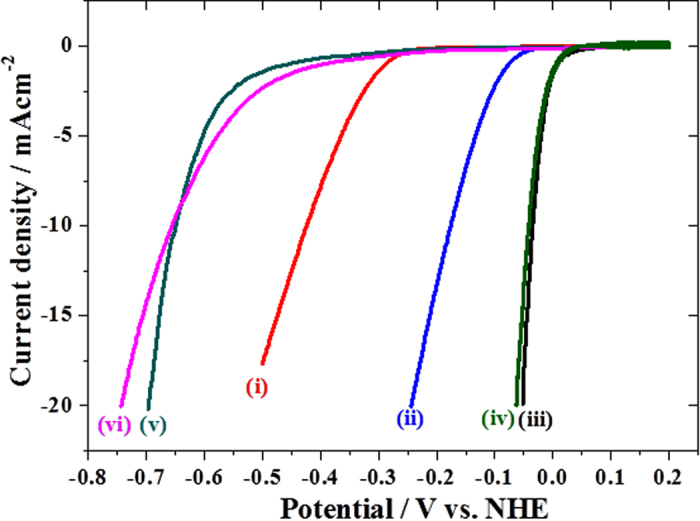
LSVs at (i) bare Au, (ii) BNNS/Au, (iii) bare Pt, (iv) BNNS/Pt, (v) Bare GC, and (vi) BNNS/GC in Ar saturated 0.5 M H_2_SO_4_ solution. Scan rate: 1 mVs^−1^.

Figure 1 shows linear sweep voltammograms (LSVs) of bare (i, iii, v) and BNNS (ii, iv, vi) modified Au (i, ii), glassy carbon (GC) (iii, iv), and Pt (v, vi) electrodes obtained in Ar saturated 0.5 M H_2_SO_4_ solution by scanning the potential negatively with 1 mV/s.

The LSV of Au electrode (i) shifts positively by ca. 250 mV by the BNNS modification (ii), showing that BNNS indeed acts as an electrocatalyst for HER at Au electrode as does for ORR, although BNNS shows negative and no effect at Pt (iii, iv) and GC (v, vi) electrodes, respectively, as observed for ORR^15^,^16^. The overpotential for HER at BNNS/Au is larger than those at Pt electrode by ca. 120 mV and 200 mV at 5 mA/cm^2^ and 15 mA/cm^2^, respectively. It is interesting to compare these values with those at transition metal dichalcogenides, which recently attract much attention as HER electrocatalyst on various substrates as mentioned above. Overpotential for HER at 15 mA/cm^2^ at WS_2_ nanosheets on GC electrodes is larger than that at Pt electrode by 200 mV^11^, which is the same as that at the BNNS/Au. MoS_2_ showed lower overpotential for HER than WS_2_ and the BNNS/Au. It is about 150 mV at amorphous MoS_2_ on GC^12^, and nanocrystalline MoS_2_ both on gold^13^ and reduced graphene oxide^14^ substrates.

To understand why BNNS modification provides Au with improved electrocatalytic activity and to find ways to further reduce the overpotential for HER, HER mechanism is considered theoretically. HER is a 2-electron process with at least two elemental steps. In acidic solution the first step is a discharge of proton as:


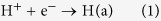


where H(a) represents hydrogen atom adsobed on an electrode surface. This process is followed by either


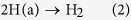


or


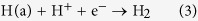


to form molecular hydrogen^4^,^5^,^6^,^7^,^8^,^9^. Since H(a) is the intermediate state, energetics of this state should play cruicial role in determining the HER rate. The importance of free eneegy/heat of adsorption of hydrogen atom on the elctrode surface was pointed out long time ago^3^,^4^,^5^ and so-called “volcano” relations between rate, or exchange current density, of HER and various forms of interaction between electrode and adsorbed hydrogen were demonstarted almost 60 years ago for the first time^4^,^5^. Recently DFT calculation was applied to obtain hydrogen chemisorption energies and volcano curve was obtained between the calculated hydrogen chemisorption energies and measured exchange current densities of HER^9^. The best electrode for HER, which situates at the top of the volcano relation, should have free energy of intermediate state, i.e., adsorbed hydrogen, close to 0 with respect to the intial state, H^+^ + e^−^, and the final state, H_2_, at equilibrium potential. Here DFT calculations are performed to determine the free energy of adsorbed hydrogen at various substrate, ΔG_H(a)_.

ΔG_H(a)_ at atoms at the terrace of free h-BN is calculated to be +2.25 eV, which is much larger than that at Au(111), which is +0.2 eV, and binding of H to the atoms at the edges of the island is very strong, i.e., too negative ΔG_H(a)_: −2.1 eV for boron atom at the edge and −2.8 eV for nitrogen atom at the edge. Thus, HER at free h-BN is not possible as H binds hardly on the terrace and too strongly at the edges. It must be noted that Nørskov *et al*. reported +0.4 eV for ΔG_H(a)_ at Au(111)^9^. This discrepancy arises from the difference in DFT functionals. While they used RPBE functional, we used WC functional because RPBE cannot reproduce BN-metal interaction.

**Figure 2:**
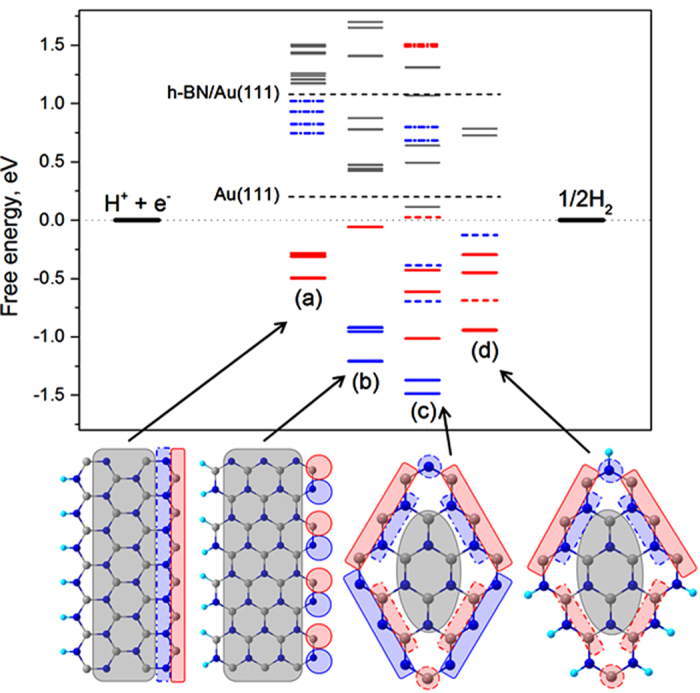
Free energy diagram for HER at equilibrium potential based on DFT calculation for BN nanoribbon (BNNR) with (**a**) zigzag and (**b**) armchair edges, (**c**) small 3 × 3 h-BN island, and (**d**) small 3 × 3 h-BN island with H-terminated nitrogen edges on Au(111) surface. Color (blue for N at edge, red for B at edge, and gray for B and N at non-edge) and symbols of lines to show ΔG_H(a)_ are matched with those indicated in the structural models shown below the free energy diagram. Optimized geometries for H(a) at these structures are shown in Supporting Information. Results of bare Au(111) and BNNS/Au(111) are also shown.

Figure 2 shows free energy diagram for HER at various 2D BN nanosystems on Au(111) at equilibrium potential based on DFT calculations. Properties of the h-BN monolayer towards H adsorption are considerably modified by Au(111) substrate. ΔG_H(a)_ at h-BN surface is decreased from +2.25 eV to +1.1 eV by being placed on Au(111), although it is still too large for HER to proceed effectively. Hydrogen adsorption behavior at the edges of BNNS islands supported on Au(111) surface is totally different from that on the extended BN surface (terrace) as shown in Fig. 2 for BN nanoribbon (BNNR) as a model of large island with (a) zigzag and (b) armchair edges, (c) small 3 × 3 h-BN island, and (d) small 3 × 3 h-BN island with H-terminated nitrogen atoms at the edge on Au(111) surface. Contrary to the bare Au(111) surface and BN terrace of extended BNNS on Au(111), these BNNS systems of finite size on Au(111) provide a large variety of non-equivalent adsorption sites. Values of ΔG_H(a)_ are dispersed widely from very negative (~ −1.5 eV) to very positive (~ +1.5 eV) and there are several sites with ΔG_H(a)_ close to 0, i.e., the thermo-neutrality condition. From close evaluation of Fig. 2, one can conclude that only atoms at the edges of BNNS islands on Au(111) can act as good electrocatalyst for HER.

Based on this theoretical consideration, further improvement of electrocatalytic activity for HER can be expected by increasing the fraction of atoms at the edges of BNNS on Au substrate. Fraction of edge atoms should be increased by decreasing the size of each BNNS at a given amount of BNNS on the surface.

**Figure 3:**
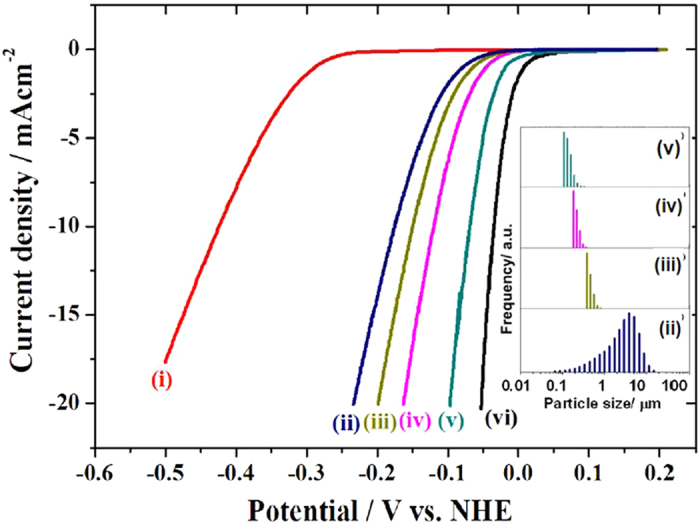
LSVs at (i) bare Au, (ii) BNNS (unfiltered)/Au, (iii) BNNS (0.45–1.0 μm)/Au, (iv) BNNS (0.22–0.45 μm)/Au, (v) BNNS (0.1–0.22 μm)/Au, and (vi) bare Pt in Ar saturated 0.5 M H_2_SO_4_ solution. Scan rate: 1 mVs−^1^. Size distribution of (ii) BNNS (unfiltered), (iii) BNNS (0.45–1.0 μm), (iv) BNNS (0.22–0.45 μm), and (v) BNNS (0.1–0.22 μm).

Figure 3 (i) and (ii) shows LSVs of bare and BNNS modified Au (BNNN/Au) electrode, respectively, as already presented in Fig. 1. In this case the size of BNNS was not controlled and is distributed from less than 0.01 μm to more than 10 μm as shown in the inset (ii). Figure 3 also shows LSVs of Au electrodes modified with BNNS of size distribution of (iii) 0.45–1.0 μm (BNNS (0.45–1.0 μm)/Au), (iv) 0.22–0.45 μm (BNNS (0.22–0.45 μm)/Au), and (v) 0.1–0.22 μm (BNNN(0.1–0.22 μm)/Au). The overpotential decreased by decreasing the size of the BNNS as expected. The best result was obtained for the Au(111) electrode modified with the smallest BNNS used in the present study (BNNS(0.1–0.22 μm)/Au). The results of overpotentials at 5 mAcm^−2^ and 15 mAcm^−2^ at various electrodes with respect to that at Pt electrode as well as exchange current densities and Tafel slopes are summarized in Table 1. The overpotential at BNNS(0.1–0.22 μm)/Au electrode is only 30 and 40 mV larger than that at Pt electrode at 5 mAcm^−2^ and 15 mAcm^−2^. These values are better than those at WS2^11^ and MoS_2_ modified electrodes^12^,^13^,^14^ as mentioned above and that at Ni_2_P on Ti substrate^24^.

Not only exchange current densities but also Tafel slopes are affected by BNNS modification. While that at bare Au electrode is 70 mV/decade, it decreases to 40 mV/decade at BNNS(unfiltered)/Au and those at BNNS(0.45–1.0 μm)/Au, BNNS(0.22–0.45 μm)/Au, and BNNS(0.1–0.22 μm)/Au are around 30 mV/decade as at Pt electrode, suggesting that HER proceeds via Volmer-Tafel mechanism. It is reasonable because there are hydrogen adsorption sites at small BN islands on Au with free energy of adsorption similar to Pt electrode^25^. Detailed mechanistic study is under way.

The stability of BNNS(0.1–0.22 μm)/Au electrode was tested by repeating the potential cycles between 0.2 V and −0.3 V. Even after 3000 cycles, overpotential was increased only 20 (at 100 mA/cm^2^) −50 mV (at 20 mA/cm^2^) as presented in Supporting Information. SEM images also presented in Supporting Information show BNNS remained on the Au surface after prolonged (5 h) HER at 20 mA/cm^2^. These results show BNNS/Au system is reasonably stable for HER.

In Summary, we have demonstrated that HER proceeds very efficiently at Au electrode, which is an inert substrate for HER, modified with BNNS, which is an insulator. Higher efficiency is achieved by using the size controlled BNNS (<1 μm) for the modification and the highest efficiency was achieved at Au electrode modified with the smallest BNNS (0.1–0.22 μm) where overpotentials were only 30 mV and 40 mV larger than those at Pt electrode at 5 mAcm^−2^ and at 15 mAcm^−2^, respectively. The Tafel slopes at Au electrode modified with size controlled BNNS were around 30 mV/decade, suggesting HER proceeds via Volmer-Tafel mechanism. DFT calculation suggests that the origin of small overpotential and Volmer-Tafel mechanism is the existence of energetically favored sites for adsorbed hydrogen, i.e., the intermediate state of HER. This work opens a new route to develop HER electrocatalysts and the development of more efficient electrocatalysts for HER is under way.

## Methods

BN powder was sonicated in IPA with 3 mg/ml as initial concentration in an ultrasonic bath for 96 h. The dispersions were centrifuged at 3000 rpm for 30 min after sonication and the 1/2 of supernatant was collected and the collected dispersion was diluted by IPA by 3 times further to be used to prepare BNNS(unfiltered)/Au electrode (1 cm x 1 cm). Size distribution of BNNS in IPA solution was determined by dynamic light scattering (DLS) method using laser scattering particle size distribution analyzer (HORIBA-LA-950V2).

Size controlled h-BNNS was obtained by filtration using MF-Millipore filter (Merck Millipore, VSWP type) of various pore size. The diluted BNNS dispersion mentioned above was filtered by a filter of 1 μm pore size filter followed by the filtration using a filter of 0.45 μm pore. The BNNS residue on the 0.45 μm filter was collected and dispersed in IPA to prepare BNNS(0.45–1.0 μm)/Au electrode. The filtrate was further filtered by a filter of 0.22 μm pore. The BNNS residue on the 0.22 μm filter was collected and dispersed in IPA to prepare BNNS(0.22–0.45 μm)/Au electrode. The filtrate was further filtered by a filter of 0.1 μm pore and the BNNS residue on the 0.1 μm filter was dispersed in IPA to prepare BNNS(0.1–0.22 μm)/Au electrode. Amount of the filtrate after the filtration using the filter of 0.1 μm pore was too small to proceed for further filtration or to use for surface modification. TEM images of the BNNS of various sizes are shown in Supporting Information.

Surface modification by h-BNNS was carried out by self-evaporation of IPA from a h-BNNS dispersion on substrates as follows. 4 to 5 gold substrates (1 cm × 1 cm) were placed perpendicularly in a 10 ml glass beaker, in which 5 ml of BNNS dispersed isopropyl alcohol (IPA) was filled. The beaker was covered by aluminum foil with small holes on the top surface and it was left at room temperature until IPA was fully evaporated (ca. 24 h) and the gold surface was covered with BNNS. The gold substrates were then heated at 120 °C in a vacuum chamber (10^−6^ Pa) for about 2 h. The gold electrode was characterized by SEM, Raman and electrochemical techniques. Raman measurements suggest majority of BNNS on Au are of monolayer as previously reported^15^.

All electrodes were pre-treated by cycling the potential between −0.1 and +1.5 V in Ar saturated 0.5 M H_2_SO_4_ electrolyte solution at a sweep rate of 100 mV s^−1^ for 100 cycles to remove any surface contaminants before the HER activity. Geometric surface area (0.5 cm^2^) was used to calculate the current density.

LSVs were recorded by varying the potential from 0.2 to −0.9 V with a scan rate of 1 mV s^−1^. All the electrochemical measurements were carried out in a 0.5 M H_2_SO_4_ an aqueous solution at room temperature. The electrolyte solution was deaerated by passing ultrapure Ar gas for at least for 1 h.

The calculations are performed using DFT with the gradient-corrected exchange-correlation functional of Wu and Cohen as implemented in the SIESTA code^15^. Computational details are given in the Supporting Information.

## Additional Information

**How to cite this article**: Uosaki, K. *et al*. Highly Efficient Electrochemical Hydrogen Evolution Reaction at Insulating Boron Nitride Nanosheet on Inert Gold Substrate. *Sci. Rep.* **6**, 32217; doi: 10.1038/srep32217 (2016).

## Supplementary Material

Supplementary Information

## Figures and Tables

**Table 1:** Summary of electrocatalytic activity of various electrodes for HER.

Electrodes	Overpotential with respect to Pt electrode		Exchange current density/A cm^−2^	Tafel slope/mVdecade^−1^
	At 5 mA cm^−2^	At 15 mA cm^−2^		
(i) Au	350	430	3.1 × 10^−7^	75
(ii) BNNS(unfiltered)/Au	110	170	1.6 × 10^−6^	40
(iii) BNNS(0.45–1.0 μm)/Au	95	130	1.4 × 10^−5^	30
(iv) BNNS(0.22–0.45 μm)/Au	70	95	2.4 × 10^−5^	28
(v) BNNS(0.1–0.22 μm)/Au	30	40	4.6 × 10^−5^	27
(vi) Pt	—	—	4.2 × 10^−4^	30
