# Supplementary material for: Highly Efficient Electrochemical Hydrogen Evolution Reaction at Insulating Boron Nitride Nanosheet on Inert Gold Substrate
Source: Sci Rep. 2016 Aug 25;6:32217. doi: 10.1038/srep32217 (PMC4997565; doi:10.1038/srep32217)
Supplement: Supplementary Information [file srep32217-s1.doc]

**Supporting Information**

Highly Efficient Electrochemical Hydrogen Evolution Reaction at Insulating Boron Nitride Nanosheet on Inert Gold Substrate

Kohei Uosaki, Ganesan Elumalai, Hung Cuong Dinh, Andrey Lyalin, Tetsuya Taketsugu, and Hidenori Noguchi

**Theoretical Methods**

The details of the calculation was described in our previous papers^1, 2^. Briefly, the calculations are performed using DFT with the gradient-corrected exchange-correlation functional of Wu and Cohen (WC)^3^ as implemented in the SIESTA package^4^. The WC functional provides an adequate description of the lattice constants, structures, and surface energies of solids and layered systems such as hexagonal boron-nitride (h-BN) monolayers deposited on 3d, 4d, and 5d transition-metal surfaces^5, 6^. Double-ζ plus polarization function (DZP) basis sets were used to treat the valence electrons of all atoms, while the core electrons were represented by Troullier-Martins norm-conserving pseudopotentials^7^. Basis set for gold was optimized with the use of the Nelder-Mead simplex method^8^. Periodic boundary conditions were used for all systems, including free molecules. The Au fcc lattice was optimized using the Monkhorst-Pack^9^ 10x10x10 k-point mesh for Brillouin zone sampling. The calculated Au lattice parameter, a = 4.1111 Å, is in excellent agreement with the experimental value of a = 4.0782 Å^10^. Optimized lattice of bulk Au was used to construct four-layer 6x6, 7x7 and 8x8 Au(111) slabs, where the bottom two layers were fixed. The 6x6 slab of gold was used as a support for the one-side H-terminated BN nanoribbon with the armchair edge, the 7x7 slab was used as a support for the one-side H-terminated BN nanoribbon with the zigzag B-terminated edge as well as for the 8x8 monolayer BN which represents gold fully covered by BN, while 8x8 slab of gold was used as a support for the bare and H-terminated 3x3 BN islands. The choice of slab sizes was stipulated by the matching conditions between the lattice parameters of the armchair- and zigzag-edged BN nanoribbons, and monolayer BN with Au(111) surface. The periodically replicated slabs were separated by a vacuum region of 20 Å. Only the Γ point was used for sampling the Brillouin zone of the slabs because of the large size of the supercell. An energy cutoff of 200 Ry was chosen to guarantee convergence of the total energies and forces. A common energy shift of 10 meV was applied. Self-consistency of the density matrix was achieved with a tolerance of 10^-4^. For geometry optimization, the conjugate-gradient approach was used with a threshold of 0.02 eV Å.

**
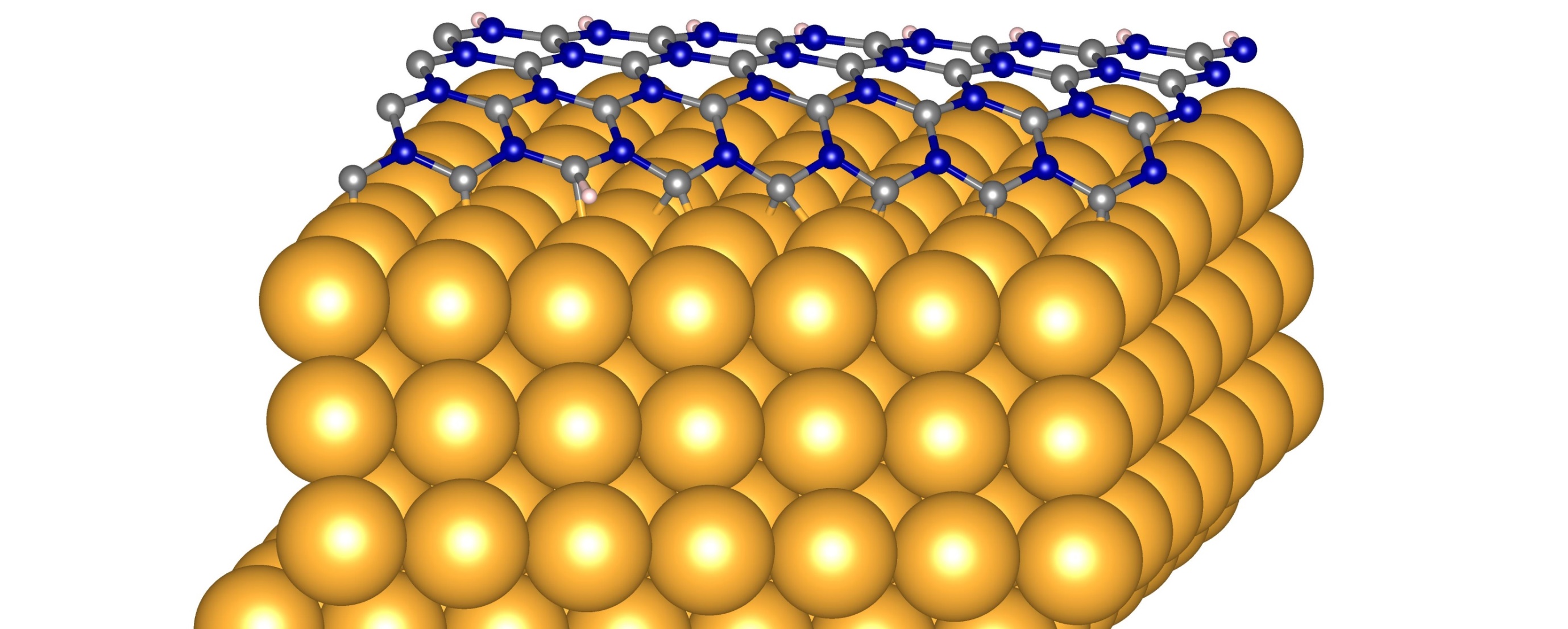
**

Figure S-1. Optimized geometry of H(a) at B of zigzag edge of BNNR on Au(111) in the regime close to thermo-neutrality.


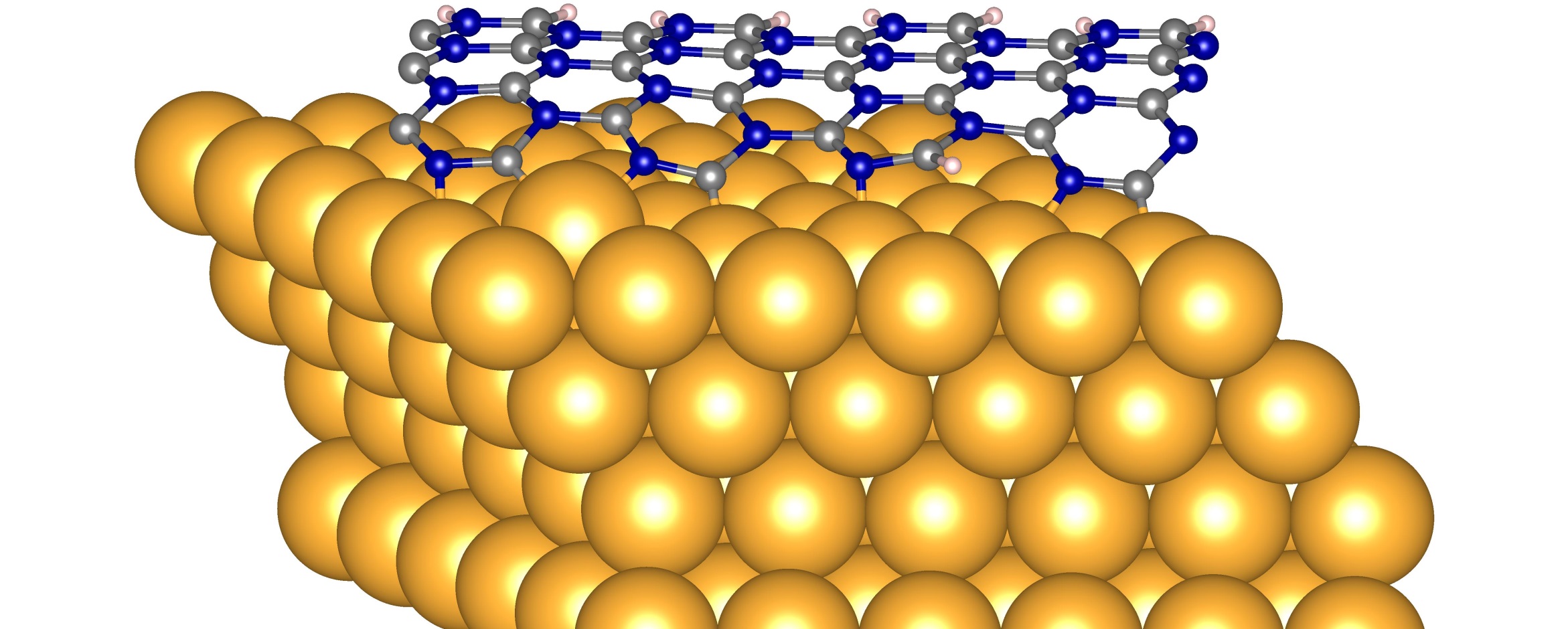


Figure S-2. Optimized geometry of H(a) at B of the armchair edge of BNNR on Au(111) in the regime close to thermo-neutrality.


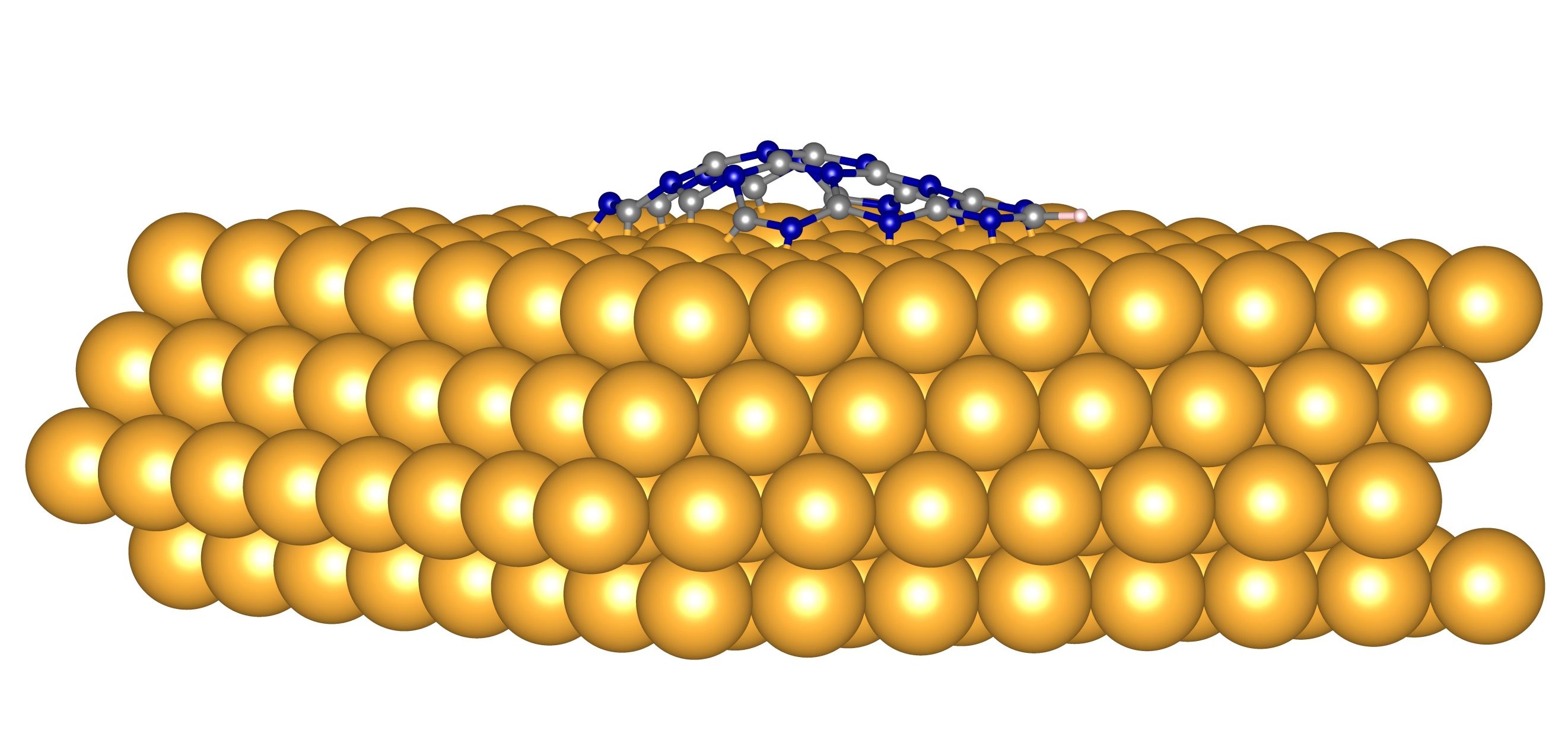


Figure S-3a. Optimized geometry of H(a) at B edge of the bare BN 3x3 island in the regime close to thermo-neutrality.


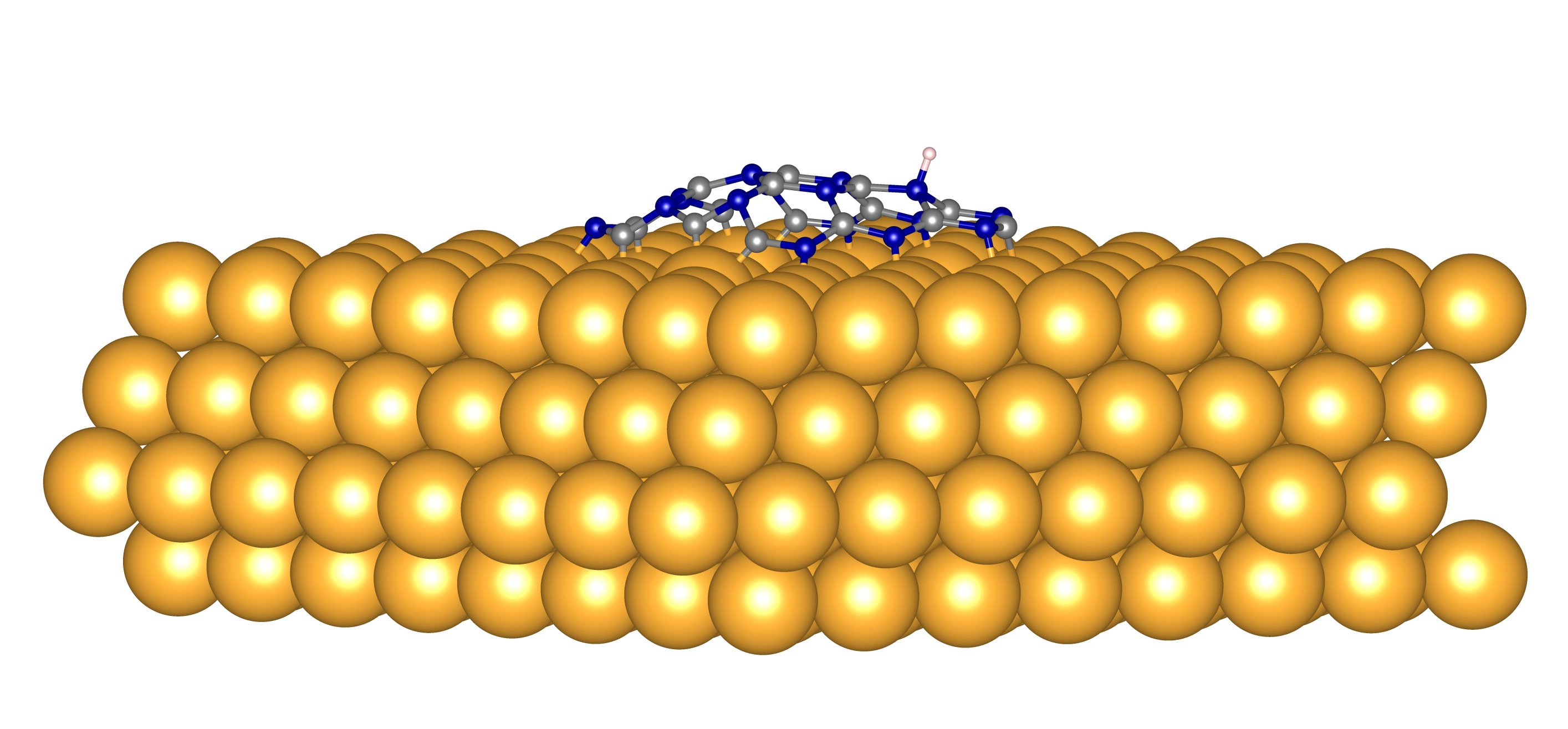


Figure S-3b. Optimized geometry of H(a) at N of the edge of bare BN 3x3 island in the regime close to thermo-neutrality.


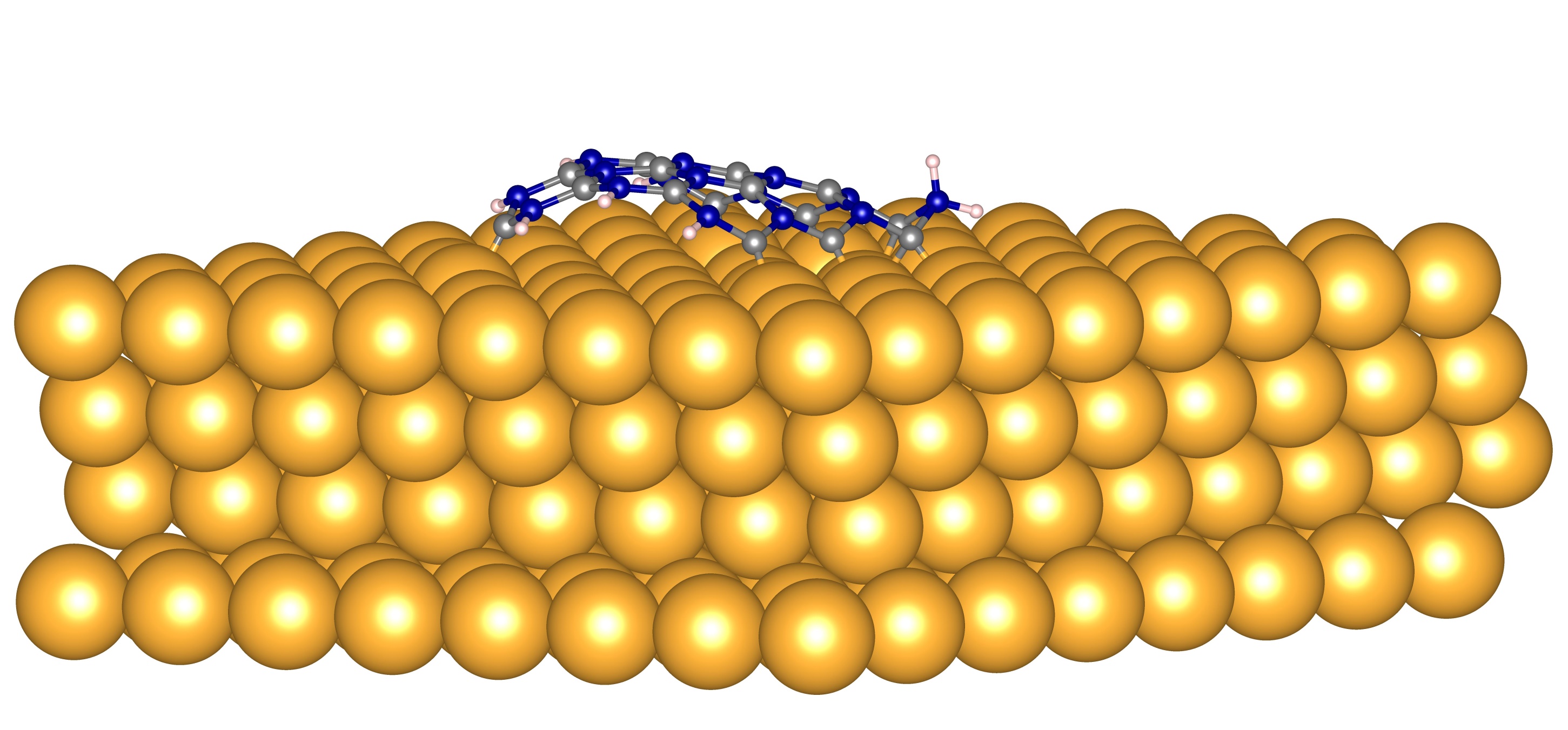


Figure S-4a. Optimized geometry of H(a) at N of the edge of BN 3x3 island with H-terminated N at the edge in the regime close to thermo-neutrality.


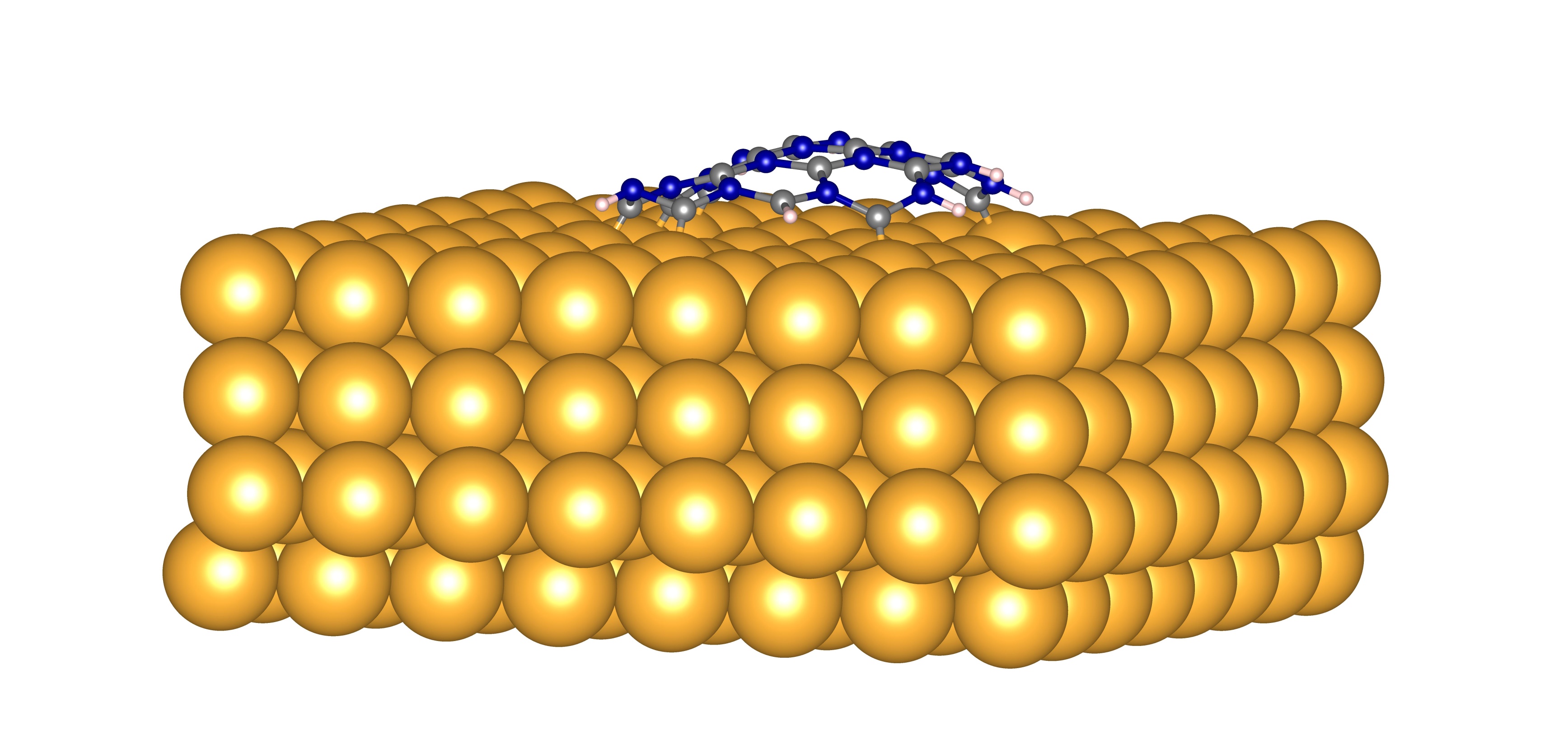


Figure S-4b. Optimized geometry of H(a) at B of the edge of BN 3x3 island with H-terminated N at the edge in the regime close to thermo-neutrality.

**TEM measurements**

Transmission electron microscopy (TEM) and High resolution (HR) TEM were carried out with a JEOL-JEM-2100F at a power of 200 keV.

TEM ((i)-(iv)) and HRTEM ((v)-(viii)) images of the liquid exfoliated^11^ BNNS of various sizes ((i) >1 µm, (ii) 0.45-1.0 µm, (iii) 0.22-0.45 µm, and (iv) 0.1-0.22 µm) are shown in Figure S-5. TEM images show that the exfoliated and filtered BN nanosheets consist of various sizes ranging from 500 nm to 100 nm (marked as red circles). TEM images also clearly shows that, when the pore size of the filter paper is controlled the size of BN nanosheets also controlled by the filtration. HRTEM images in Figure S-5((v)-(viii)) shows that the BNNS consists of single to few layers with honeycomb lattice structure (hexagonal atomic structure) and the fast Fourier transform (FFT) image shown in fig. 2(c) inset confirms that BNNS is composed of hexagonal atomic structure. Few layer (> 3layers) sheets mainly observed at >1 µm, and 0.45-1.0 µm size filters whereas at 0.22-0.45 µm, 0.1-0.22 µm filters observed only single to 3 layers of BN nanosheets. It clearly says that the pore size of the filters controls not only the BN size but also the thickness or layers of each BN sheets also controlled. Zigzag and armchair edge structures^12^ are clearly observed in Figure S-5((v)-(viii)).


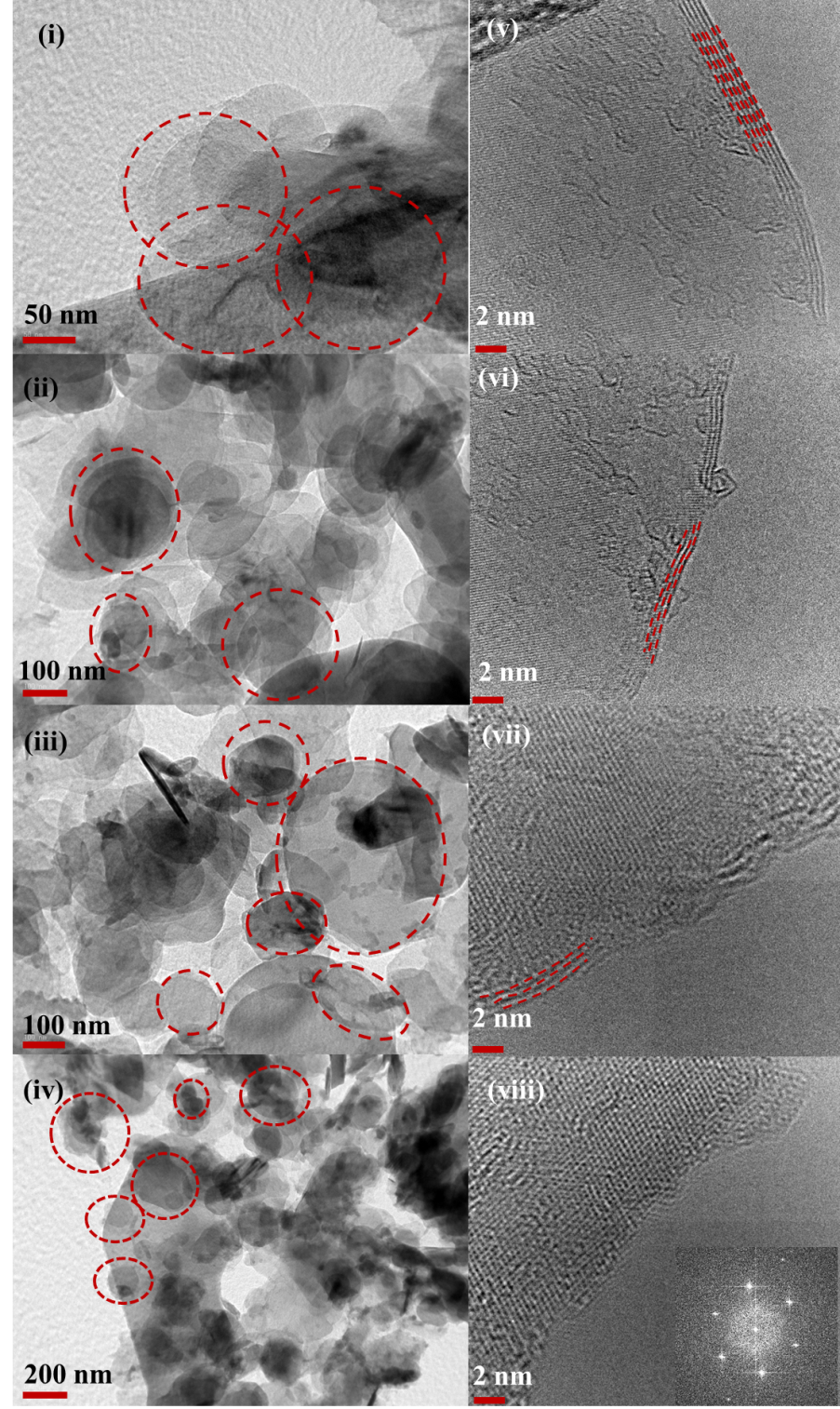


Figure S-5. TEM ((i) – (iv)) and HRTEM images ((v)-(viii)) of the liquid exfoliated BNNS with various size distribution: (i) >1 µm, (ii) 0.45-1.0 µm, (iii) 0.22-0.45 µm, and (iv) 0.1-0.22 µm. Inset of (viii) is the fast Fourier Transform of the image.

**SEM measurements**

Scanning electron microscopy (SEM) was carried out by Hitachi, FE-SEM S-4800 at a power of 5 keV.

Figure S-6 shows SEM images of BNNS(0.1-0.22 µm)/ Au (a) before and (b) after HER with 20 mA/cm^2^ for 5h. BNNS (black spots) were uniformly distributed throughout the gold substrate in both cases. The sizes of exfoliated BNNS are between 200 to 400 nm in the lateral dimension as reported before.^14^ The presence of BNNS on Au even after the long HER (Fig. 2(b)) confirms that the BNNS strongly adsorbed on gold substrate.

**
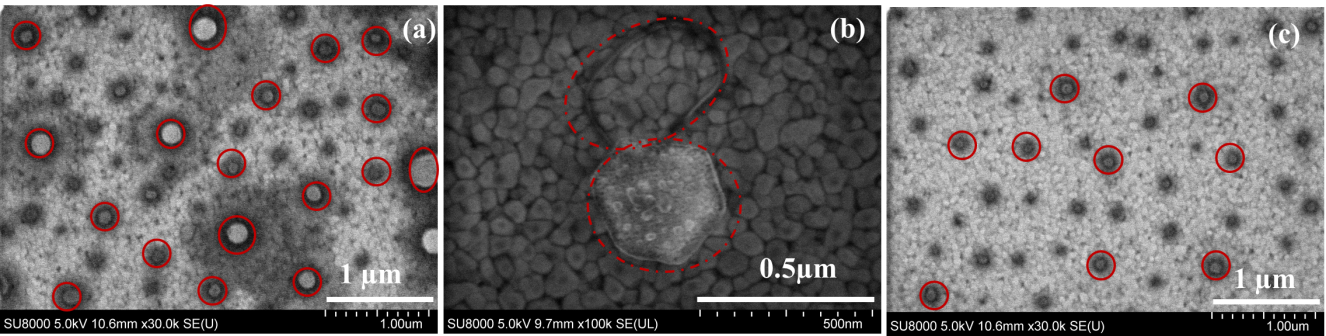
**

Figure S-6: SEM images of BNNS(0.1-0.22 µm)/Au (a) before and (b) after HER.

**Stability Test**

Figure S-7 shows polarization curves of BNNS(0.1-0.22 µm)/Au electrode between 0.2 V and -0.3 V in Ar saturated 0.5 M H_2_SO_4_ solution with the scan rate of 50 mV/sec for the 1st scan and after 3000 scans. 20 mA cm^-2^ flowed at -105 mV initially but at 155 mV, i.e., 50 mV larger overpotential, after 3000 cycles. For the current density of 100 mA cm^-2^, the potential difference between the initial and after 3000 cycles was only 20 mV. These results show the BNNS/Au system is reasonably stable for HER. current density of 20 mA cm^-2^ flowed at -105 mV initially but at 155 mV, i.e., 50 mV larger overpotential, after 3000 cycles. For the current density of 100 mA cm^-2^, the potential difference between the initial and after 3000 cycles was only 20 mV.

**
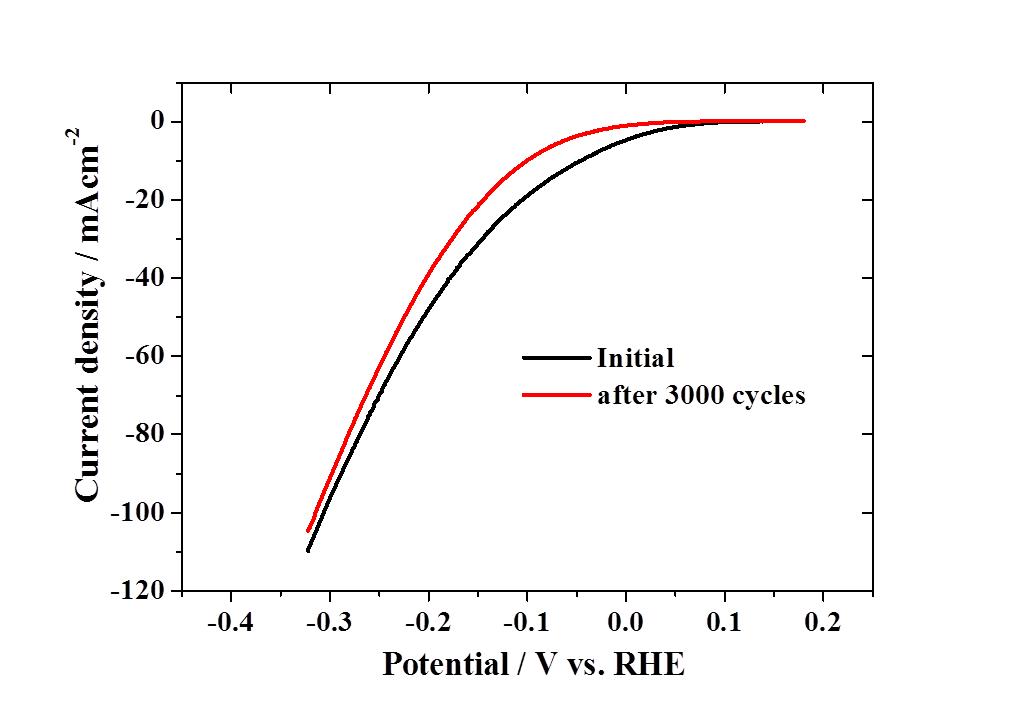
**

**Figure 2:** Polarization curves at BNNS(0.1-0.22 µm)/Au electrode in Ar saturated 0.5 M H_2_SO_4_ solution with the scan rate of 50 mV/sec.

References

1. Lyalin, A., Nakayama, A., Uosaki, K. & Taketsugu, T. Theoretical predictions for

hexagonal BN based nanomaterials as electrocatalysts for the oxygen reduction reaction. *Phys. Chem. Chem. Phys.* **15,** 2809-2820 (2013).

1. Lyalin, A., Nakayama, A., Uosaki, K. & Taketsugu, T. Functionalization of monolayer h-BN by a metal support for the oxygen reduction reaction. *J. Phys. Chem. C* **117,** 21359-21370 (2013).
2. Wu, Z. & Cohen, R. E. More accurate generalized gradient approximation for solids. *Phys. Rev. B: Condens. Matter Mater. Phys.* **73,** 235116 (2006).
3. Soler, J. M., Artacho, E., Gale, J. D., García, A., Junquera, J., Ordejón, P. & Sánchez-Portal, D. The SIESTA method for ab initio order – *N* materials simulation. *J. Phys.: Condens. Matter.* **14,** 2745-2779 (2002).
4. Tran, F., Laskowski, R., Blaha, P. & Schwarz, K. Performance on molecules, surfaces, and solids of the Wu-Cohen GGA exchange – correlation energy functional. *Phys. Rev. B: Condens. Matter Mater. Phys.* **75,** 115131 (2007).
5. Laskowski, R., Blaha, P. & Schwarz, K. Bonding of hexagonal BN to transition metal surfaces: An ab initio density – functional theory study. *Phys. Rev. B: Condens. Matter Mater. Phys.* **78,** 045409 (2008).
6. Troullier, N. & Martins, J. L. Efficient pseudopotentials for plane – wave calculations. *Phys. Rev. B: Condens. Matter Mater. Phys.* **43,** 1993–2006 (1991).
7. Nelder, J. A. & Mead, R. A simplex method for function minimization. *The Computer Journal* **7,** 308–313 (1965).
8. Monkhorst, H. J. & Pack, J. D. Special points for Brillouin – zone integrations. *Phys. Rev. B* **13,** 5188-5192 (1976).
9. Maeland, A. & Flanagan, T. B. Lattice spacings of gold – plalladium alloys. *Can. J. Phys.* **42,** 2364-2366 (1964).
10. Coleman, J. N. et al. Two – dimensional nanosheets produced by liquid exfoliation of layered materials. *Science* **331,** 568-571 (2011).
11. Alem, N., Erni, R., Kisielowski, C., Rossell, M. D., Gannett, W. & Zettl, A. Atomically thin hexagonal boron nitride probed by ultrahigh – resolution transmission electron microscopy. *Phys. Rev. B* **80,** 155425 (2009).
12. Gorbachev, R. V. et al. Hunting for monolayer boron nitride: Optical and Raman signatures. *Small* **7,** 465 – 468 (2011).
13. Uosaki, K. et al. Boron nitride nanosheet on gold as an electrocatalyst for oxygen reduction reaction: Theoretical suggestion and experimental proof. *J. Am. Chem. Soc.* **136,** 6542-6545 (2014).
